# Preparing for Transcription: The Role of Histone H2A.Z

**DOI:** 10.1371/journal.pbio.0030413

**Published:** 2005-11-01

Every cell in our body contains the instructions for life encoded in around two meters of DNA. In eukaryotic cells (cells with nuclei), all this DNA is squeezed into the cell's nucleus, a region about one-hundredth of a millimeter across. Cells accomplish this improbable task with the help of histones. These proteins combine with DNA to form chromatin, which is made up of structural units called nucleosomes. Nucleosomes, in turn, consist of about 146 base pairs of DNA wrapped around an eight-unit structure containing two molecules each of four core histones—H2A, H2B, H3, and H4. Each nucleosome is separated from its neighbors by a short “linker” DNA. This “beads-on-a-string” arrangement folds into a smooth fiber, which folds into thicker fibers so that all the DNA packs neatly into the nucleus.

Unfortunately, this tidy solution renders chromatin-packaged DNA mostly inaccessible to the transcription machinery. Consequently, cells have devised several ways to adjust the position and/or characteristics of nucleosomes to allow gene expression, including the incorporation of variant histones into nucleosomes. The evolutionarily conserved histone variant H2A.Z (also called Htz1 in yeast) is implicated in transcriptional regulation and gene silencing (inactive, or silent, genes are packaged into dense chromatin called heterochromatin), but little is known about how H2A.Z, which replaces H2A in some nucleosomes, regulates these biological functions. Knowing exactly where in the genome H2A.Z takes the place of H2A should provide insights into how this particular variant histone regulates genome structure and function—which is why Benoît Guillemette and colleagues set out to map H2A.Z binding sites throughout the yeast genome.

The researchers used a technique called chromatin immunoprecipitation to isolate DNA sequences bound to specific histones in living yeast cells, then amplified them, and determined their position in the yeast genome using microarray analysis. They report that H2A.Z binds to 4,862 small regions (which they call Z loci) scattered across the yeast genome. 74% of these regions lie over promoters, regulatory DNA sequences at the start of transcribed genes; 63% of yeast promoters are decorated with H2A.Z. The authors show that H2A.Z specifically associates with one or two nucleosomes within the promoter of some inactive genes but is generally absent from promoters of highly active genes. In addition, they provide evidence that the chromatin structure is more organized in terms of exact positioning of the nucleosomes and that H2A.Z may play a role in that promoter-specific chromatin organization.

The authors also describe the physical pattern of binding of H2A.Z to a second type of region in the genome—Htz1-activated domains (HZADs). These domains are found in euchromatin (loosely packed, actively transcribed chromatin) lying next to heterochromatin, and it is thought that H2A.Z binding stops the spread of silencing into euchromatin, a function called antisilencing. The researchers report that H2A.Z occupies a wider region around HZAD genes than it does at Z loci, indicating that H2A.Z may affect gene transcription and antisilencing through different mechanisms.

Overall, the authors propose that H2A.Z has two roles in yeast cells: to poise genes for transcription initiation in euchromatin and to protect euchromatin from silencing. Whether H2A.Z incorporation into nucleosomes is necessary and sufficient for these activities, and whether it has additional transcriptional effects in yeast and other organisms, is not yet known, but this map of H2A.Z binding sites in the yeast genome will be invaluable in future investigations into the mechanisms of gene regulation. —*Jane Bradbury*

